# MooSciTIC: Training of trainers in West African research and higher education

**DOI:** 10.1371/journal.pbio.3000312

**Published:** 2019-06-07

**Authors:** Ménonvè Atindehou, Kifouli Adéoti, Laura Estelle Yêyinou Loko, Thierry Beulé, Emmanuel Paradis, Gustave Djedatin, Christine Tranchant-Dubreuil, François Sabot, Latifou Lagnika, Estelle Jaligot

**Affiliations:** 1 Laboratoire de Biochimie et Substances Naturelles Bioactives, Faculté des Sciences et Techniques, Université d'Abomey-Calavi, Cotonou, Bénin; 2 LAMITA, Faculté des Sciences et Techniques, Université d'Abomey-Calavi, Cotonou, Bénin; 3 Université Nationale des Sciences, Technologies, Ingénierie et Mathématiques (UNSTIM), Abomey, Bénin; 4 CIRAD, UMR DIADE, Montpellier, France; 5 DIADE, Univ Montpellier, IRD, Montpellier, France; 6 ISEM, Univ Montpellier, CNRS, IRD, EPHE, Montpellier, France

## Abstract

The MooSciTIC project is a capacity-building initiative targeting West African research scientists and higher education teachers. The project aimed to improve the self-reliance of researchers and upgrade research practices by providing on-site summer schools on trans-disciplinary topics such as scientific writing, communication, and integrity. Here, we explain how this program was designed and implemented and share the positive responses from our trainees, hoping to inspire similar initiatives.

## Abstract 2

This Community Page article describes the MooSciTIC project, which aims to allow West African scientists to improve their skills in scientific writing, communication and ethics, while fostering new collaboration opportunities.

## Rationale and aim of the MooSciTIC project

In our experience, the training of young researchers and teaching assistants in French-speaking Western African countries often lacks proper courses on key research-oriented aspects such as literature search, scientific writing, project management, scientific integrity, or ethics [1,2]. In France and other high-income countries such cross-cutting notions are typically provided to postgraduate students through on-the-job training with varying degrees of supervision. This early training enables students to be progressively involved in "real life" research work through projects and publications and to start building their professional network. By contrast, mentoring from experienced colleagues is not always available at institutions in low-income countries, resulting in a wide variation in research quality and visibility [3]. This is especially true in French-speaking West Africa where research is highly fragmented and collaborative work culture is lacking, including at the national and regional levels.

**Fig 1:**
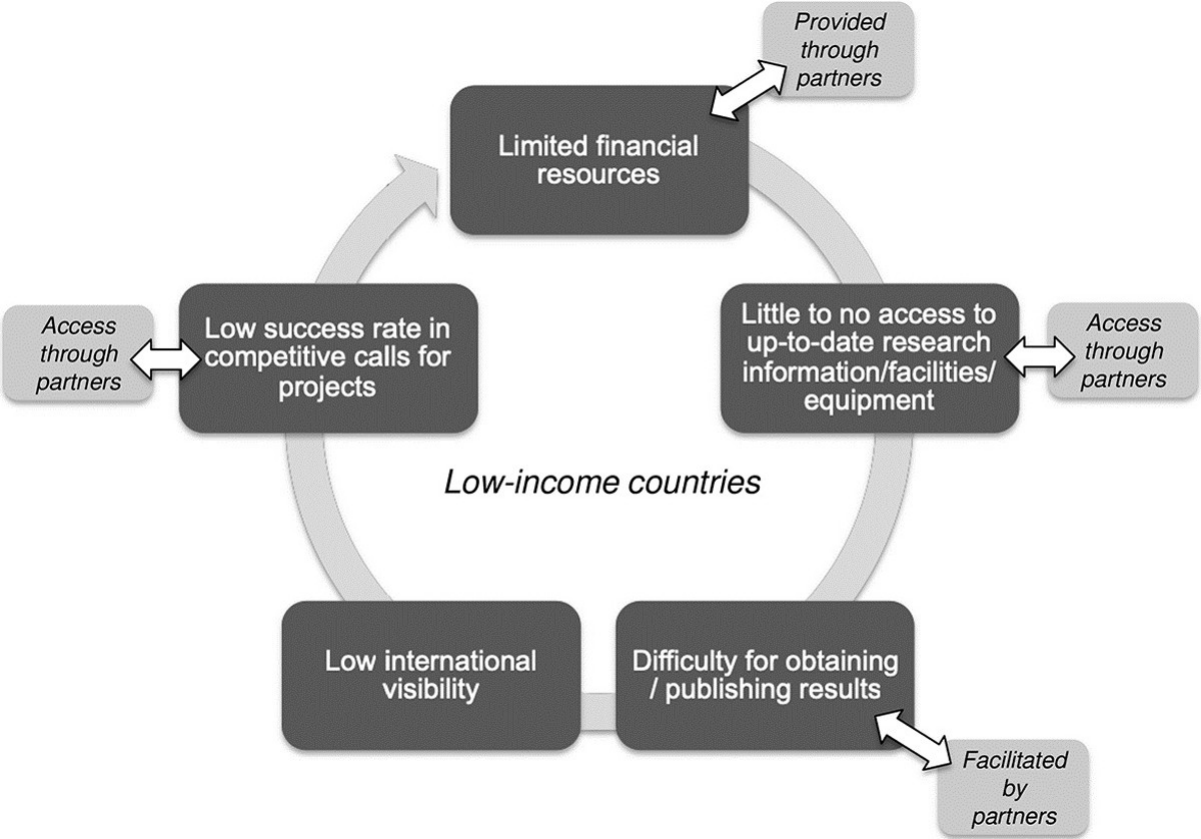
The vicious circle of research cooperation between low- and high-income countries. Several steps of the circle can be partially compensated through cooperation with high-income countries, while bringing no solution to the central issue (i.e., lack of resources), creating dependency to the collaboration.

This skill gap, we feel, might be one element of a vicious circle (Fig 1) reminiscent of the Matthew effect [4], describing how successful science and scientists tend to become more successful with time. In this case, scientists from low-income countries are locked into continued dependency on their partners from high-income countries for the access to both high-quality publications and competitive funding (allocated by mostly North-based entities), which ultimately do not always benefit research priorities of the South [2,5–8]. Sub-Saharan Africa (minus South Africa) accounts for less than 1% of the world's scientific publications and is even less represented among peer-reviewed ones [9–11]. African scientists, like researchers from other developing countries, are more likely to fall prey to "predatory publishers" due to strong institutional pressure that rewards quantity over quality of publications [12,13].

**Fig 2:**
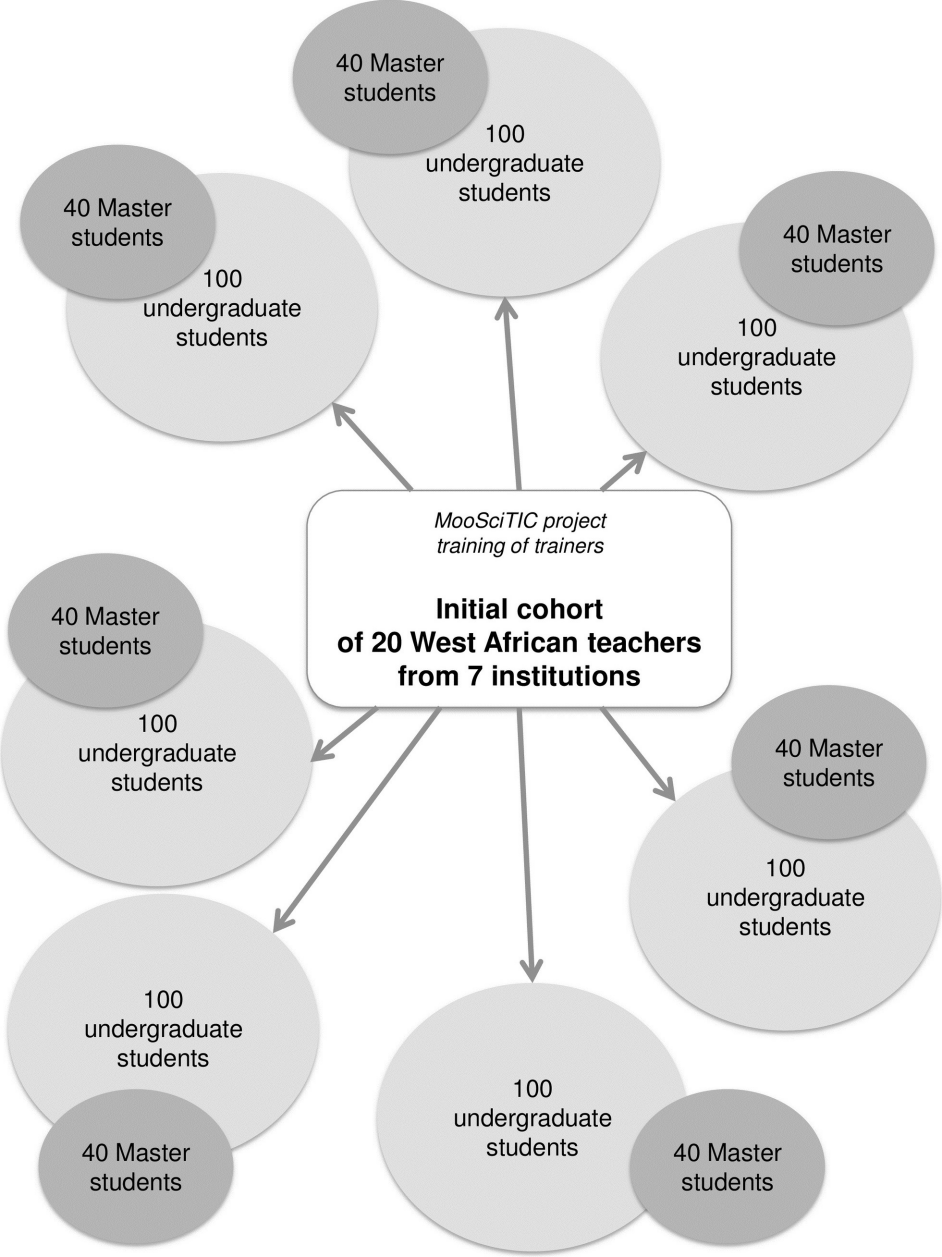
Expected impact of the MooSciTIC project. For clarity, this figure only shows the impact-enhancing effect of the training of a hypothetical cohort of teachers on average numbers of undergraduate and master students within 1 academic year.

In this context, we designed the "MooSciTIC: A shot of science!" project as a small-scale capacity-building initiative aimed at West African teaching assistants, early-career lecturers, and research scientists, focusing on cross-disciplinary aspects of their work. In order to maximize the long-term impact of our training, we used a "training of trainers" format: based on a rough estimate provided by West African colleagues from different universities of the subregion, we anticipated that each teacher would teach to 40 master students and an even greater number of undergraduate students. Throughout the 3 years of the project, we could therefore expect to indirectly reach hundreds of students (Fig 2). We anticipated a further amplifying effect over the duration of each teacher's career, and out of the habit, common in West Africa, of disseminating one's training among peers within one’s home institution. Though similar issues arise in English-speaking Africa, we targeted this geographical and linguistic subregion due to a history of collaboration between research institutes in France and French-speaking Western Africa.

## Training design and implementation

**Fig 3:**
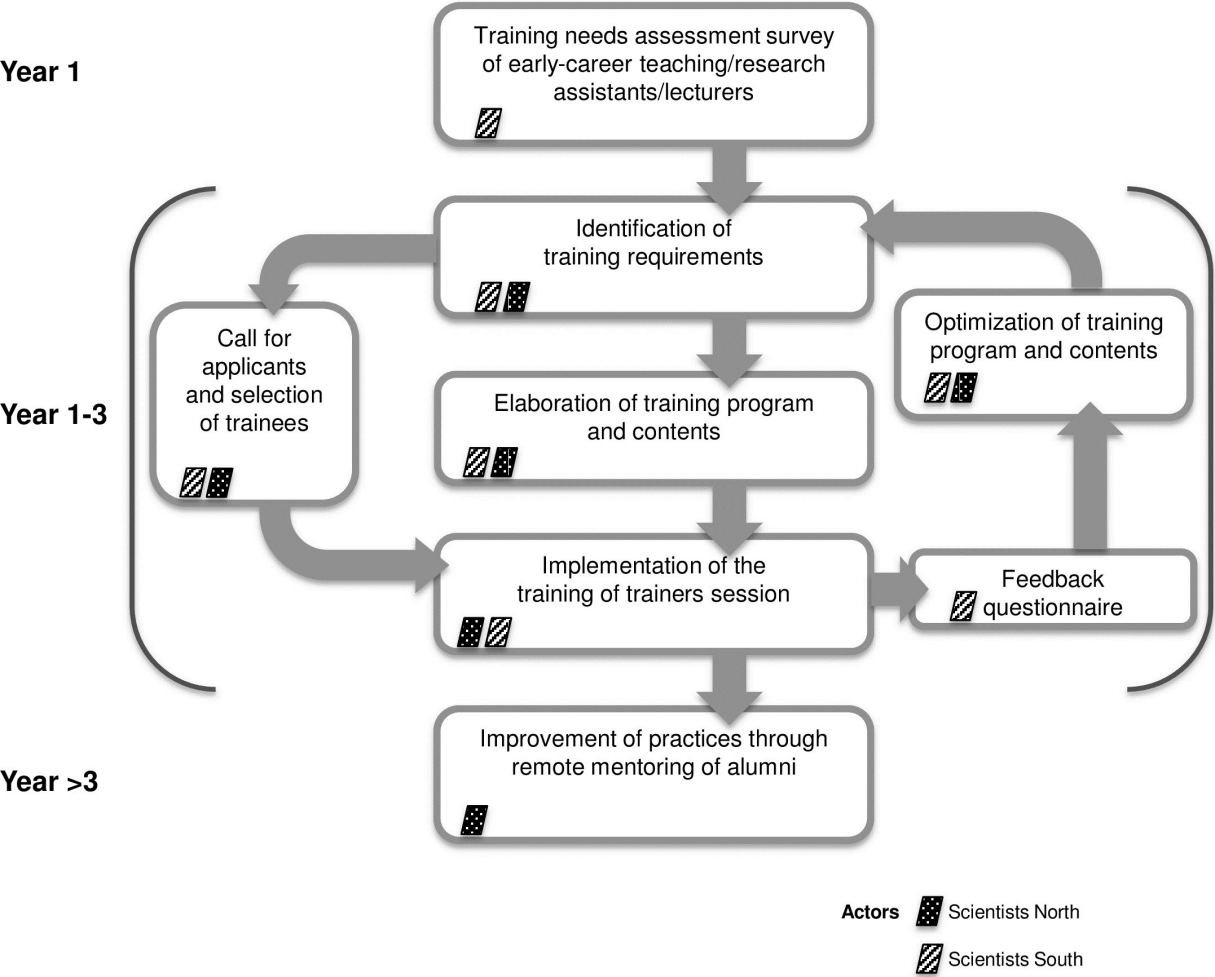
Structure of the MooSciTIC project.

The overall structure of the MooSciTIC project is illustrated in Fig 3.

An initial needs assessment survey was conducted in order to define the training requirements of West African scientists and teachers (S1 Data, "Survey" tab). Because we aimed to provide knowledge and materials that could be instantly reused by our trainees in their daily work and disseminated to peers and students, we selected active (participative and collaborative) learning methods for their demonstrated higher efficiency for such purposes [14,15]. We conceived the program so that each topic would be addressed through sections combining traditional lectures and group and/or class activities in order to favor both the acquisition of on-the-job experience and emulation (Table 1). Wherever applicable, we demonstrated the use of popular freeware tools, institutional repositories, social platforms, and online resources made freely available to developing countries, so that the future implementation of these activities would not be contingent on access to paywalled items.

**Table 1:** Program of the MooSciTIC summer school.

Topic	Contents
**Literature mining and reference database management**	Theory: • Metadata organization and use; • Principles of web-based literature search: queries and search engines; Practice/interactivity: • Reference management tools: main functionalities.
**Developing, funding, and managing a project**	Theory: • Organizing ideas and formalizing a project (mind/concept mapping); • Answering a call for projects (deciphering terms of reference, eligibility criteria; developing scientific, temporal and financial aspects of the project; anticipating funders' and reviewers' expectations); • Project management. Practice/interactivity: • Demonstration of mind/concept mapping tools; • Demonstration of project/task management tools; • Responding to a fictional call for projects (group activity); • Individual feedback on a personal draft project.
**Scientific communication**	Theory: • Basic principles for writing an article (building the backbone: material and methods, figures and tables; finding the core message: trimming experimental data; parallel structuration of discussion and introduction; citations practices; navigating the editorial process and anticipating evaluation criteria); • Tips for improving oral presentations and slideshows; • Making an efficient poster presentation; • The scientific resume for grant and job applications. Practice/interactivity: • Individual feedback on personal draft articles and posters; • Oral presentation of fictional projects.
**Mechanisms of scientific investigation**	Practice/interactivity: • Case-based activity "An inexplicable disease."
**Scientific integrity and research ethics**	Theory: • Basic concepts and principles of research integrity; • Illustrations: real-life cases; • Ethics in publishing (predatory publishers) and reviewing. Practice/interactivity: • Testimonials and debate.
**Societal concerns in research**	Practice/interactivity: • Testimonials and debate around a societal question of interest for research.

Call for applicants were advertised through e-mails to partner institutions and institutional representations throughout the subregion. In order to achieve the desired amplifying effect, we selected participants from as many countries and institutions as possible. Achieving gender parity among trainees was challenging: with applications from female scientists amounting to a third (81/243) of total applications, we had no other choice than to use positive discrimination as a corrective measure. As shown in S1 Fig, a fair proportion of the gender, geographical, and institutional diversity among applicants was successfully preserved in our selection.

Training sessions were hosted by the University of Abomey-Calavi (UAC) in Cotonou, Bénin, in 2016, 2017, and 2018. We opened each sequence related to scientific writing skills for either articles or grant proposals with a course recapitulating cardinal rules, because a large proportion of rejections of submitted documents may be attributed to insufficient compliance to writing and organization guidelines, regardless of the scientific quality of the contents [16,17]. We provided practical tips for improving writing efficiency and avoiding common pitfalls in the reviewing process, based on both our experience and several popular "how to" writing manuals [18–21].

In order to quickly give trainees opportunities to put principles into practice, we ensured that the different parts of the program were connected by an underlying theme. We usually provided sections on literature search and grant proposal writing during the first day and then gave trainees a fictional call for projects of broad impact in life sciences (for instance, "Biodiversity assessment and preservation") to work on throughout the summer school. Groups of 4 to 5 people were then required to choose from several options and build up a grant proposal made of a 1-page abstract, a budget table, and a Gantt chart fitting within the constraints of the call. Later in the program, we provided a course on principles of good oral presentations so that trainees would be well prepared for the final presentation of their project to the class. A few selected project titles are provided as an illustration (S1 Table).

During the set-up of the first summer school, we found the paper from Justin Hines and colleagues [22] describing their use of a simulation sequence named "An inexplicable disease" for teaching the basic principles of scientific investigation through role play. We thought the activity’s elegant simplicity and versatility might be interesting to our trainees and as a new teaching tool they might use, so we included it to our program. As an unexpected bonus, we observed that it was also efficient for revealing both group dynamics and individual characters among trainees so that we used it with great success as an “ice breaker” on the first day of the 2018 summer school.

We felt it was important to include societal concerns regarding science in the program, because their influence on how research is implemented is becoming increasingly important. Reflecting our commitment to gender equity, we selected "African women in research" as the subject of a debate in 2016, with local guests providing testimonies. In 2017 and 2018, we invited local competent authorities to discuss the important consequences that the progressive enforcement of the Nagoya Protocol on Access and Benefit Sharing (ABS) [23] will have on the distribution of powers in North–South research collaborations. Another societal focus was inspired by the increasing public attention gathered by recent headline-grabbing stories of scientific misconduct and fraud. Such news has triggered a global realization that research integrity training is insufficient and needs upgrading [24–26]. Our course on scientific integrity was initially built on examples of misconduct and proved difficult for the trainees to relate to. In the following years, we improved it by adding daily-life situations illustrating both good and questionable reseach practices.

We distributed our teaching materials under a Creative Commons license to facilitate reuse. Together with complementary resources pertaining to the summer school, they were provided to trainees on thumb drives at the end of each session.

## Feedback from the trainees

In the last days of each session, we provided a questionnaire to the participants in order to probe their perception of the training and improve future sessions. Briefly, the global clarity and consistency of the program was rated as "good" or "very good/excellent" by a large majority of the respondents (83.3% to 100% depending on the year), with a continuous increase of the latter appreciation over time (from 22.2% in 2016 to 65.2% in 2018). The aspects of the program that were the most appreciated and had the highest potential for immediate reuse were those relative to project development and management, scientific communication, and bibliography. Further quantitative analyses are available in S1 Data ("Post-session feedback" tab). Additional lessons learned are summarized in Box 1.

**Box 1. Take-home points.**

**1. Choose your (teaching) niche and stick to it**

At the onset of the project, we were not confident that a training made exclusively of interdisciplinary topics would attract enough interest. Therefore, science-oriented contents were included in the first session, making up half of the program. However, trainees felt that this attempt at casting too wide a net left them with insufficient time to explore each subject in any depth. To us, it became clear that the risk of losing our audience to frustration far outweighed the benefits from diversifying topics. These "miscellaneous" contents were therefore cut by half in 2017, then dropped in 2018. As we narrowed down of our scope to what was its core concept, satisfaction rates raised accordingly.

**2. Not everything is about teaching; leave ample time for interactions**

The group activity around the fictional call for project that was woven into the fabric of the MooSciTIC summer school required us to "save" large time slots of 2 to 3 consecutive hours each day, so that trainees could research and work together. As shown in the feedback, this was also highly appreciated as an opportunity for professional networking.

**3. Aiming to please everyone on all aspects, all the time is futile**

Many of the suggestions we received for improvements of the summer school amounted to mutually incompatible or unrealistic propositions. For instance, daily work schedules were generally considered to be too heavy, yet the same persons would suggest that more time should be dedicated to each topic. The emphasis we put on international scientific communication highlighted how much the lack of English proficiency is a major obstacle to the visibility of research from francophone West Africa [6]. This prompted suggestions that "English as a foreign language" should be included to the program, although no significant improvement in language skills could be achieved in our short time frame. We made choices, explained them, and encouraged our trainees to seek complementary training.

**4. Be mindful of external factors; obstacles are not always what you expect**

Some of the issues raised in the feedback were relative to points over which we had little to no control but that ended up having a significant influence. Many comments revolved around the fact that we could not provide financial compensations for expenses incurred by trainees. This was a pervasive problem throughout the project because the scarcity of mobility grants both led to a large proportion of withdrawals among selected applicants (S1 Fig) and left most participants with no other option than to self-fund while, in some cases, their salary had been suspended. Our decision to reduce the duration of the summer school partly addressed this issue. Additionally, we worked out an agreement with a local caterer to ensure that, in addition to their lunch, trainees could take away food for their dinner. Part of the success of 2016 and 2017 sessions, which coincided with Ramadan, can be attributed to this measure.

We assessed the longer-term impacts of the training by asking participants from the 2016 and 2017 editions to respond to another questionnaire (S1 Data, "Delayed feedback" tab). Twenty (59%) responded, of which 90% reported improved oral communication and presentation skills; 70% increased efficiency and quality in research publication; 60% improved student supervision; 40% increased success in competitive grant applications. Forty percent mentioned that they had reused our teaching materials to train students and/or fellow scientists, the most frequently cited being scientific integrity and ethics. Finally, 25% of respondents associated their latest tenured position or promotion to learning outcomes from MooSciTIC.

## Conclusion

We believe our experience with the MooSciTIC summer school demonstrates the relevance of capacity building through training of trainers in the specific context of West Africa. We hope our experience inspire other teachers from the North and the South to engage in similar initiatives, hopefully with a clearer vision of their opportunities and pitfalls. As part of our teaching material, we provided all of our trainees with the documents that had been submitted for funding under the MooSciTIC project, as a case in point for successful project development and writing. We see the present paper as another opportunity to illustrate how their work and ours can be promoted and generate added value for the wider scientific community.

True capacity building implies a shift in ownership and leadership [27,28], and thus it is now up to our West African partners and alumni to take over and disseminate the MooSciTIC initiative further. However, no lasting transformation can be achieved without commitment from governments and local policy makers [1,2]. Their willingness to provide incentives for boosting the training of scientists, developing research infrastructures through planned investment, and favoring the emergence of coordinated national and transnational research networks is key for securing an independent future for West Africa.

## Ethics statement

Surveys, application forms, and questionnaires included disclaimers stating that collected data would only be used after anonymization and for the purpose of project assessment only. Gender parity was enforced consistently throughout the project among coordinators, teachers, and trainees. All teaching material produced and/or used as part of this project is distributed (in French) under the Creative Commons license Attribution–NonCommercial–ShareAlike (BY-NC-SA) 4.0 International (http://creativecommons.org/licenses/by-nc-sa/4.0/) and is available upon request. In time, this material will be made available on the project's website (http://mooscitic.ird.fr/).

## Supporting information

S1 Data Numerical data supporting the present article. Data underlying the initial needs assessment survey (Survey), applicant selection according to geographical and institutional origin and gender (S1 Fig, panels A, B and C), self-assessments of the training at the end of each session (Post-session feedback) and 1 or 2 years afterward (Delayed feedback), respectively, are displayed in separate tabs of this file.
(XLSX)

S1 Fig Geographical, institutional, and gender diversity among total applicants, selected applicants, and participants to the MooSciTIC training sessions. Data are displayed separately for each of the 3 years of the project: 2016 (A), 2017 (B), and 2018 (C). Selected applicants were included in the initial selection list, whereas participants correspond to selected applicants minus withdrawals plus replacements from the waiting list. M, F indicate male and female applicants, respectively. Higher education and research institutions are numbered as follows: 1: Centre Inter-Facultaire de Formation et de Recherche en Environnement pour le développement Durable (CIFRED, UAC); 2: Ecole Nationale Supérieure des Sciences et Techniques Agronomiques, Djougou; 3: Ecole Polytechnique d’Abomey-Calavi; 4: Ecole Nationale d'Economie Appliquée et de Management (ENEAM, UAC); 5: Faculté des Sciences et Techniques (FAST), Dassa-Zoumè; 6: Institut CERCO (private university); 7: Institut National de l'Eau (INE, UAC); 8: Institut Régional de Santé Publique Comlan Alfred Quenum, Ouidah (UAC/WHO); 9: Commission Nationale du Développement Durable; 10: Univ. Abomey-Calavi (UAC), Cotonou; 11: Univ. Parakou; 12: AfricaRice (CGIAR Consortium Research Center); 13: Institut National De La Jeunesse De L'éducation Physique Et Du Sport (INJEPS), Univ. Porto-Novo; 14: Institut du Développement Rural (Univ. Polytechnique Bobo-Dioulasso); 15: Univ. Ouaga I Prof. Joseph Ki-Zerbo, Ouagadougou; 16: Univ. Polytechnique Bobo-Dioulasso; 17: Faculté d'Agronomie et de Bioingénierie, Univ. Burundi; 18: École Nationale Supérieure des Sciences Agro Industrielles, Univ. Ngaoundéré; 19: Univ. Bamenda; 20: Univ. Yaoundé; 21: Univ. Kinshasa (UNIKIN); 22: Centre de Recherches Oceanologiques (CRO), Abidjan; 23: Institut National Polytechnique Félix Houphouët-Boigny (INP-HP), Yamoussoukro; 24: Univ. Félix Houphouët-Boigny; 25: Univ. Jean Lorougnon Guédé, Daloa; 26: Univ. Nangui Abrogoua, Abidjan; 27: Univ. Péléforo Gon Coulibaly, Korhogo; 28: Univ. Tahoua; 29: Univ. Tillabéri; 30: Ecole Supérieure de Génie Industriel et Biologique, Dakar; 31: Institut Sénégalais de Recherche Agricole (ISRA), Dakar; 32: Univ. Assane Seck, Ziguinchor; 33: Univ. Cheikh Anta Diop (UCAD), Dakar; 34: Univ. Lomé; 35: African School of Economics; 36: Centre de Recherche sur le Paludisme Associé à la Grossesse et à l'Enfance (CERPAGE); 37: Faculté des Sciences Agronomiques (FSA, UAC); 38: Faculté des Sciences de la Santé (FSS, UAC); 39: Faculté des Sciences et Techniques (FAST, UAC); 40: Université Nationale des Sciences, Technologies, Ingénierie et Mathématiques (UNSTIM), Abomey; 41: Institut National de la Recherche Agronomique du Bénin, Centre de Recherches Agricoles—Plantes Pérennes (INRAB-CRAPP); 42: Université Nationale d'Agriculture, Kétou; 43: Institut de recherche en sciences appliquées et technologies; 44: Univ. Koudougou; 45: Univ. Alassane Ouattara; 46: Centre d’Etude Régional pour l’Amélioration de l’Adaptation à la Sécheresse (CERAAS); 47: Ecole Normale Supérieure (ENS) de Natitingou; 48: Ecole supérieure Le Faucon, Abomey-Calavi; 49: Univ. Inter Régionale du Génie Industriel des Biotechnologies et Sciences Appliquées (IRGIB)—Africa; 50: Centre International de Recherche-Développement sur l’Elevage en zone Subhumide (CIRDES); 51: Univ. Fada N'Gourma; 52: Univ. Gbadolite (UNIGBA); 53: Univ. Man (U-Man); 54: Institut Supérieur de Formation Agricole et Rurale (ISFAR), Univ. Thiès; 55: Ecole Supérieure des Techniques Biologiques et Alimentaires (ESTBA), Univ. Lomé; 56: Institut Togolais de Recherche Agronomique (ITRA), Lomé; 0: no affiliation. Note that affiliations to different substructures within the same complex institution (e.g., UAC) are indicated whenever possible depending on the information provided by applicants. DRC, Democratic Republic of the Congo.
(TIF)

S1 Table Examples of project outlines. (DOCX)

## Abbreviations

ABS: Access and Benefit Sharing
UAC: University of Abomey-Calavi
