# Supplementary figures and images for: MooSciTIC: Training of trainers in West African research and higher education
Source: PLoS Biol. 2019 Jun 7;17(6):e3000312. doi: 10.1371/journal.pbio.3000312 (PMC6583953; doi:10.1371/journal.pbio.3000312)

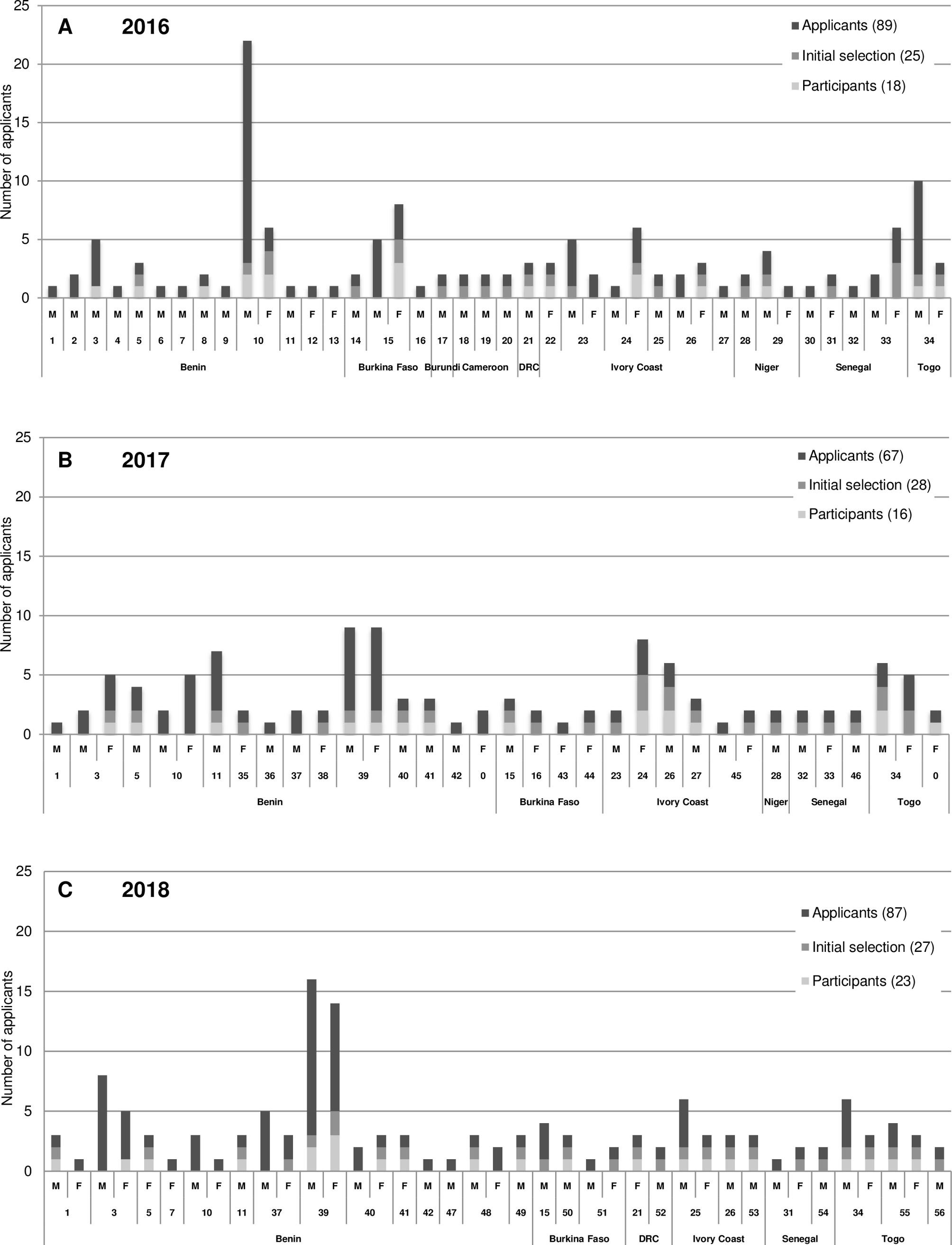

Supplement: S1 Fig — Data are displayed separately for each of the 3 years of the project: 2016 (A), 2017 (B), and 2018 (C). Selected applicants were included in the initial selection list, whereas participants correspond to selected applicants minus withdrawals plus replacements from the waiting list. M, F indicate male and female applicants, respectively. Higher education and research institutions are numbered as follows: 1: Centre Inter-Facultaire de Formation et de Recherche en Environnement pour le développement Durable (CIFRED, UAC); 2: Ecole Nationale Supérieure des Sciences et Techniques Agronomiques, Djougou; 3: Ecole Polytechnique d’Abomey-Calavi; 4: Ecole Nationale d'Economie Appliquée et de Management (ENEAM, UAC); 5: Faculté des Sciences et Techniques (FAST), Dassa-Zoumè; 6: Institut CERCO (private university); 7: Institut National de l'Eau (INE, UAC); 8: Institut Régional de Santé Publique Comlan Alfred Quenum, Ouidah (UAC/WHO); 9: Commission Nationale du Développement Durable; 10: Univ. Abomey-Calavi (UAC), Cotonou; 11: Univ. Parakou; 12: AfricaRice (CGIAR Consortium Research Center); 13: Institut National De La Jeunesse De L'éducation Physique Et Du Sport (INJEPS), Univ. Porto-Novo; 14: Institut du Développement Rural (Univ. Polytechnique Bobo-Dioulasso); 15: Univ. Ouaga I Prof. Joseph Ki-Zerbo, Ouagadougou; 16: Univ. Polytechnique Bobo-Dioulasso; 17: Faculté d'Agronomie et de Bioingénierie, Univ. Burundi; 18: École Nationale Supérieure des Sciences Agro Industrielles, Univ. Ngaoundéré; 19: Univ. Bamenda; 20: Univ. Yaoundé; 21: Univ. Kinshasa (UNIKIN); 22: Centre de Recherches Oceanologiques (CRO), Abidjan; 23: Institut National Polytechnique Félix Houphouët-Boigny (INP-HP), Yamoussoukro; 24: Univ. Félix Houphouët-Boigny; 25: Univ. Jean Lorougnon Guédé, Daloa; 26: Univ. Nangui Abrogoua, Abidjan; 27: Univ. Péléforo Gon Coulibaly, Korhogo; 28: Univ. Tahoua; 29: Univ. Tillabéri; 30: Ecole Supérieure de Génie Industriel et Biologique, Dakar; 31: Institut Sénégalais de Recher [file pbio.3000312.s002.tif]
