# Supplementary material for: MooSciTIC: Training of trainers in West African research and higher education
Source: PLoS Biol. 2019 Jun 7;17(6):e3000312. doi: 10.1371/journal.pbio.3000312 (PMC6583953; doi:10.1371/journal.pbio.3000312)
Supplement: S1 Table — (DOCX) [file pbio.3000312.s003.docx]

**S1 Table: Examples of project outlines.**

| **Year** | **Project type ^a^** | **Theme** | **Budget size** |
| --- | --- | --- | --- |
| 2016 | A | Identification of drought- and heat-tolerant genotypes of yam through molecular markers, for enhanced resilience to climate change. | 50 k€ |
| 2016 | B | Assessment of mangrove biodiversity for improved resource management and preservation. | 500 k€ |
| 2017 | B | Impact of gold panning on biodiversity and identification of restorative measures. | 500 k€ |
| 2018 | A | Charaterization of the genetic and biochemical diversity of plant species used as food wrapping. | 50 k€ |
| 2018 | B | Influence of anthropic pressure on hippopotamus populations. | 500 k€ |

^a^ Available project types in this fictional call are:

A = Initial biodiversity characterization and inventory. Duration: 2 years.

B = Assessment of the impact of human activities on biodiversity (maximum range: last century). Duration: 3-4 years.

C = Analysis of long-term biodiversity evolution. Duration: 3-4 years.
